# Supplementary material for: Effects of Grazing, Extreme Drought, Extreme Rainfall and Nitrogen Addition on Vegetation Characteristics and Productivity of Semiarid Grassland
Source: Int J Environ Res Public Health. 2023 Jan 5;20(2):960. doi: 10.3390/ijerph20020960 (PMC9859310; doi:10.3390/ijerph20020960)
Supplement: Supplementary file 1 [file ijerph-20-00960-s001.zip › ijerph-1993604-supplementary.pdf]

Supplement of

Effects of Grazing, Extreme Drought, Extreme Rain-Fall and Nitrogen Addition on Vegetation Characteristics and Productivity of Semi-Arid Grassland

Table S1 Variance analysis of the response of soil moisture content and soil nitrogen content to grazing, rainfall and nitrogen addition in sandy grassland

Treatment	Soil moisture content / (%)		Soil nitrogen content / (%)	
	F	Sig.	F	Sig.
Grazing	8.159	0.005	0.026	0.873
rainfall	0.313	0.868	1.604	0.179
nitrogen	0.117	0.732	3.664	0.058
Grazing × rainfall	1.044	0.388	1.594	0.182
Grazing × nitrogen	0.013	0.909	0.426	0.516
Rainfall × nitrogen	0.183	0.947	0.945	0.441
Grazing × rainfall × nitrogen	0.339	0.851	0.590	0.671

Table S2 Correlation between vegetation characteristics and soil water and nitrogen content in sandy grassland

	Coverge / (%)	Species richness	Plant total density	Litter biomass / ($g.m^{-2}$)	Simpson dominance index	Shannon-Wiener diversity index	Pielou evenness index	Aboveground biomass / ($g.m^{-2}$)	soil moisture content / (%)	soil nitrogen content / (%)
CWM_{height}	0.335**	0.033	0.061	0.038	-0.093	0.075	.101	0.642**	0.131	-0.089
Coverge / (%)		0.102	0.154	0.038	-0.216*	0.179	0.191*	0.411**	0.033	-0.032
Species richness			-0.076	-0.065	-0.559**	0.816**	-0.021	0.145	0.054	-0.070
Plant total density				-0.052	0.040	-0.051	0.014	0.103	-0.026	0.236**
Litter biomass / ($g.m^{-2}$)					0.198*	-0.175	-0.240**	0.120	0.101	0.095
Simpson dominance index						-0.922**	-0.789**	-0.180*	0.059	0.136
Shannon-Wiener diversity index							0.547**	0.171	-0.030	-0.110
Pielou evenness index								0.136	-0.140	-0.130
Aboveground biomass / ($g.m^{-2}$)									0.183*	-0.004
soil moisture content / (%)										-0.012

Note: * indicates significant correlation between two variables ($p < 0.05$), ** indicates extremely significant correlation between two variables ($p < 0.01$).
